# Barriers to Health Equity and Contributors to Health Disparities Among Individuals with Intellectual and Developmental Disabilities: A Narrative Review

**DOI:** 10.3390/future4020012

**Published:** 2026-03-24

**Authors:** Ebele Okoye, Jerome Bronson, Mary Shaw, Robyn Breland, Angela Omondi

**Affiliations:** 1 Department of Epidemiology & Biostatistics, College of Health Sciences, Jackson State University, Jackson, MS 39213, USA; 2 Mississippi Delta Center of Excellence in Maternal Health, Jackson, MS 39213, USA; 3 Department of Health Policy & Management, College of Health Sciences, Jackson State University, Jackson, MS 39213, USA; 4 Department of Behavioral & Environmental Health, College of Health Sciences, Jackson State University, Jackson, MS 39213, USA; 5 Special Olympics Mississippi, Jackson, MS 39216, USA

**Keywords:** access to healthcare, developmental disabilities, health inequities, health disparities, healthcare barriers, intellectual disabilities, structural barriers

## Abstract

**Background:**

Individuals with intellectual and developmental disabilities (IDD) experience persistent health disparities that result in poorer health outcomes, reduced quality of life, and inequitable access to healthcare.

**Objective:**

This narrative review synthesized existing literature to identify key barriers to health equity and contributors to health disparities among individuals with IDD.

**Method:**

This study was a narrative (non-systematic) review that adopted a qualitative synthesis approach. A literature review was conducted across PubMed, CINAHL, PsycINFO, Medline, and Google Scholar to identify peer-reviewed articles published between 2010 and 2025 that address health disparities, health inequities, healthcare barriers, and social determinants of health among individuals with IDD. Thematic analysis was employed to synthesize the included studies and identify recurring patterns and themes.

**Results:**

A total of 88 articles were included. Two overarching domains shaping health disparities were identified: barriers to health equity and contributing factors. Seven barrier categories emerged: attitudinal, communication, policy, programmatic, social, physical, and transportation. Five key contributors were also identified: limited access to healthcare, comorbid conditions, low health literacy, adverse social determinants of health, and caregiver burden.

**Conclusions:**

Health disparities among individuals with IDD are driven by intersecting social, structural, and healthcare system barriers rather than individual limitations alone. This review informs policymakers, public health professionals, and interventionists on how to advance health equity for individuals with IDD through targeted, person-centered interventions.

## 1. Introduction

Intellectual and developmental disabilities (IDD) are typically present at birth or arise during early childhood and are characterized by limitations in intellectual functioning, adaptive behavior, and/or physical, language, learning, or behavioral development that persist throughout the lifespan [1]. Individuals with IDD experience greater health disparities compared to the general population, which is reflected in higher rates of mortality, greater morbidity, and poorer overall quality of life [2]. These inequities extend beyond health outcomes to include patterns of healthcare access and utilization, where individuals with IDD encounter persistent challenges securing timely, appropriate, and equitable care [2,3].

Epidemiological study estimates further emphasize the public health significance of this population. Globally, the prevalence of intellectual disability (ID) is generally considered to be between 1 and 2% of the general population, though estimates vary widely based on methodological and demographic differences [4]. Maulik et al. [4] reported a pooled global prevalence of 10.37 per 1000 individuals in a meta-analysis of studies from 52 countries, noting significant variation by country income level, study population age group, and study design. In the United States, developmental disabilities are common among children, and their prevalence has risen in recent years. Approximately 17% of children are affected by developmental disabilities, with prevalence increasing over the past two decades due to rising diagnoses of autism, attention-deficit/hyperactivity disorder (ADHD), and intellectual disability [1,5]. Recent national estimates indicate that approximately 5.8% of Americans across all ages, racial groups, and socioeconomic strata live with an intellectual disability [6].

Adults with IDD face a disproportionate burden of chronic health conditions, including obesity, asthma, diabetes, cardiovascular disease, chronic pain, epilepsy, and mental health disorders, contributing to substantial disparities throughout the lifespan [7,8]. However, these disparities do not arise solely from biological or developmental risks. Rather, they reflect the cumulative effects of disadvantages structured by social and economic conditions. Children and adults with developmental disabilities (DD) face significantly higher rates of unmet social needs (like housing, food, and healthcare access) and health-related social needs (HRSNs) compared to peers, experiencing roughly 2.5 times the odds of needing help with basic needs, even after accounting for demographics, indicating systemic gaps in care. Factors like low income, racial/ethnic background, and communication barriers often worsen these disparities, affecting access to therapy, mental health support, and daily resources, necessitating better screening and integrated support in healthcare systems [9,10]. The interaction of these social risks with communication challenges, reduced health literacy, and limited access to disability-competent care further amplifies inequities and restricts opportunities for early intervention, prevention, and chronic disease management.

Research studies reported disparities in healthcare access and utilization for individuals with IDD, leading to delayed diagnosis, fragmented care, limited availability of specialized providers, lengthy waiting times, and significant transportation and communication barriers [11–14]. Studies also reported that structural and environmental constraints, along with policy gaps and workforce limitations, exacerbate inequitable health outcomes for this population [15,16]. Although these findings have increased awareness of health disparities, the lived experiences and multilayered challenges faced by individuals with IDD remain underrepresented in public health research, policy design, and systems-level interventions [17]. Previous studies emphasize that advancing health equity requires strengthening provider training, increasing access to inclusive services, improving communication support, enhancing care coordination, and implementing disability-informed policies and health promotion strategies [18–20]. However, much of the existing literature focuses on isolated barriers or single health outcomes within narrow contexts. Despite widespread recognition of inequities affecting this population, few studies offer a comprehensive synthesis that integrates the barriers to health equity and the broader contributors that intensify health disparities. To address this gap, this narrative review synthesized the existing literature on key barriers to health equity and the underlying contributors to health disparities among individuals with IDD. The study findings will inform policymakers and public health practitioners in designing tailored, individual-centered interventions for this population.

## 2. Materials and Methods

This study was a narrative (non-systematic) review that adopted a qualitative synthesis approach to provide a comprehensive overview of health disparities and barriers to health equity among individuals with IDD. A literature search was conducted using Boolean operators across the following databases: PubMed, CINAHL, PsycINFO, Medline, and Google Scholar. Search terms included intellectual disability, developmental disability, IDD, health disparities, health equity, barriers to care, health literacy, healthcare access, healthcare utilization, and provider attitudes. Searches were limited to articles published in English between 2010 and 2025. Relevant peer-reviewed studies examining health disparities, healthcare barriers, and health outcomes among children, adolescents, or adults with IDD were included. Additional articles were identified through the reference list review of selected studies. Conference abstracts, theses, studies without accessible full text, and articles not focused on individuals with IDD were excluded. Most of the included studies were conducted in high-income countries, reflecting the available evidence. Articles were reviewed, and findings were synthesized to identify recurring patterns and key themes across the literature.

## 3. Results

A total of 88 articles were included in this narrative review, encompassing both qualitative and quantitative studies. Of these, 45 addressed barriers to health equity, while 43 focused on contributors to health disparities among individuals with IDD. The review of the literature revealed two overarching domains: (i) barriers to achieving health equity embedded within healthcare systems and broader social structures, and (ii) factors contributing to persistent health disparities among individuals with IDD.

**Figure 1.**
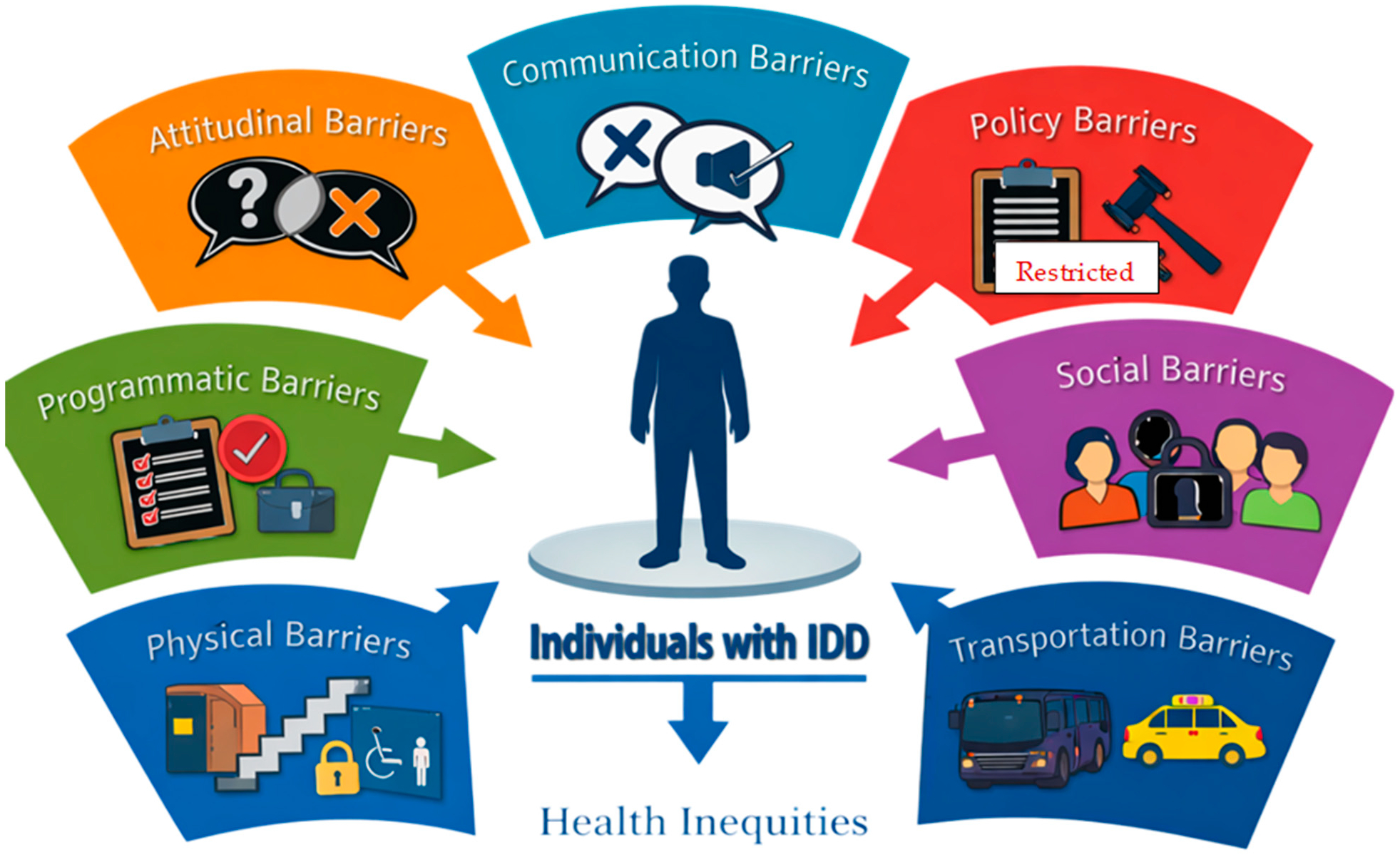
Barriers to health equity among individuals with IDD.

**Figure 2.**
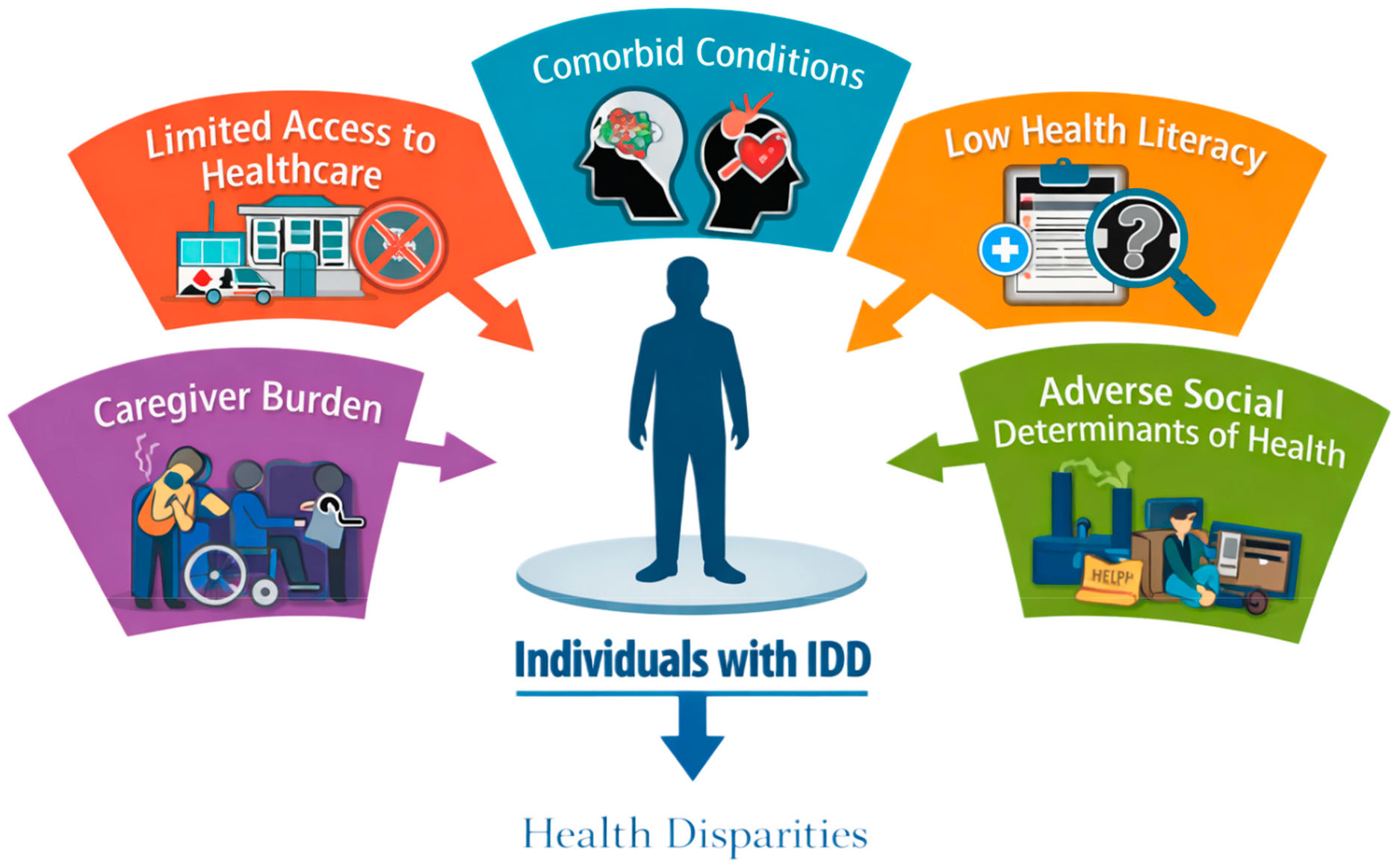
Factors contributing to health disparities among individuals with IDD.

Across the literature, seven recurring categories of barriers were identified: attitudinal, communication, policy, programmatic, social, physical, and transportation barriers (Figure 1). Attitudinal and communication barriers were most frequently reported across healthcare settings. In addition, five key contributors to health disparities were consistently identified: limited access to healthcare, comorbid conditions, low health literacy, caregiver burden, and adverse social determinants of health (SDOH), shown in Figure 2. These factors were often described as interconnected and mutually reinforcing, contributing to inequities across the life course of individuals with IDD.

## 4. Discussion

This narrative review indicates that health disparities among individuals with IDD arise from both persistent barriers to health equity and broader social and structural contributors to poor health outcomes. The findings reveal that limitations within healthcare systems, such as inadequate access to disability-competent care, ineffective communication, and fragmented services, function as direct barriers to equitable care. At the same time, social and economic conditions, including poverty, comorbidity, transportation challenges, and caregiver burden, contribute to the persistence and severity of these disparities. The following sections provide a more detailed discussion of these themes and their interrelationships.

### 4.1. Barriers to Health Equity Among Individuals with IDD

#### 4.1.1. Attitudinal Barriers

Findings from this narrative review indicate that attitudinal barriers represent a foundational determinant shaping healthcare experiences and outcomes for individuals with IDD. These barriers stem from persistent societal misconceptions and negative beliefs that portray individuals with IDD as having a diminished quality of life, limited capabilities, or inherently poor health [21]. Such attitudes are often expressed through stereotypes, prejudice, and paternalistic assumptions, such as pity or perceptions of incompetence, that contribute to exclusion and discrimination across healthcare, employment, and social contexts. These attitudes frequently arise from limited disability awareness and a tendency to conceptualize disability as an individual deficit rather than as a consequence of societal and structural barriers. As a result, individuals with IDD may experience infantilization, denial of hidden disabilities, intrusive behaviors such as staring, or provider discomfort rooted in fear of “doing the wrong thing” [22]. At the provider level, attitudinal barriers have been identified as a major impediment to primary care access among individuals with IDD [11]. Negative provider attitudes have long contributed to stigmatization and misdiagnosis within healthcare systems. Fisher and Purcal [23] report that such perceptions often marginalize individuals with disabilities by framing their health concerns as inherent to their disability rather than as indicators of treatable conditions. This stigmatization increases the risk of diagnostic overshadowing, delays in care, and inadequate treatment, ultimately worsening health outcomes. Consistent with this pattern, diagnostic overshadowing, where new or treatable symptoms are misattributed to disability rather than appropriately investigated, remains one of the most consequential manifestations of attitudinal bias [24]. These assumptions obscure comorbid conditions, delay intervention, and narrow treatment options by reducing clinical vigilance. Prior research has further shown that negative provider attitudes can undermine patient autonomy and dignity. Biased perceptions among healthcare professionals are associated with substandard care, reduced access to preventive services, and poorer health outcomes for individuals with IDD [24,25]. Hotez et al. [26] emphasize that stigmatizing attitudes embedded within clinical environments perpetuate structural inequities, reinforcing healthcare spaces that are unwelcoming, inaccessible, or dismissive of disability-related needs. These dynamics are often exacerbated by organizational pressures, such as time constraints and productivity demands, which encourage reliance on stereotypes rather than on individualized assessment. Provider characteristics and institutional culture also shape the persistence of attitudinal barriers. Evidence suggests that factors such as staff age, training background, and exposure to person-centered models of care influence perceptions of disability. For example, higher staff age has been associated with more negative views toward person-centered care and informal support practices [27], emphasizing the importance of ongoing training and organizational commitment to disability-inclusive care.

#### 4.1.2. Communication Barriers

Communication barriers also emerged in this narrative review as a critical impediment to equitable healthcare for individuals with IDD. These barriers arise from both individual communication needs and systemic failures within healthcare environments that prevent them from being accommodated effectively. Many individuals with IDD rely on alternative communication methods, such as visual supports, gestures, assistive devices, or simplified language, which are often unrecognized or inadequately integrated into clinical practice [28]. High rates of communication difficulties have been documented among adults with intellectual disabilities, with approximately 75% using verbal speech yet still facing substantial challenges when interacting with healthcare providers and caregivers [28]. These difficulties are associated with ID severity, low social participation, challenging behaviors, and conditions such as Down syndrome. Importantly, communication challenges significantly affect quality of life by limiting self-determination, social inclusion, and emotional well-being. When communication needs are unmet, individuals with IDD may struggle to articulate symptoms, understand medical instructions, or provide informed consent, increasing the likelihood of missed diagnoses, inappropriate treatment decisions, and reduced engagement in care [29,30]. These challenges can range from mild to profound and directly influence the ability to engage meaningfully in healthcare encounters. Provider-level factors further exacerbate communication barriers. Poor communication by healthcare providers (HCPs) has been shown to create significant barriers to quality care for individuals with learning disabilities (LDs). Inadequate communication practices, often resulting from failure to use alternative methods such as Makaton or visual aids, can lead to dehumanizing care experiences, clinical errors, and substandard care [31]. These shortcomings contribute to frustration, disempowerment, and diminished engagement among individuals with IDD and their caregivers. Additionally, many healthcare providers lack training in disability-informed communication, resulting in rushed clinical encounters or communication directed primarily to caregivers rather than directly to patients with IDD [32]. Such practices undermine patient autonomy, limit shared decision-making, and increase the risk of diagnostic errors and misinterpretation of symptoms, ultimately contributing to inequitable care and poorer health outcomes [33]. Educational gaps among nurses and other healthcare professionals further compound these challenges, as limited disability-focused training and experiential learning opportunities reduce confidence and reinforce reliance on stereotypes [34]. Consistent with prior studies, system-level constraints also play a substantial role. Time pressures within clinical environments frequently limit opportunities for meaningful communication, leaving individuals with IDD without sufficient support to process health information or ask questions McIlfatrick et al. [35]. Environmental and structural shortcomings further contribute to communication inequities, as healthcare settings rarely provide accessible materials, such as plain-language documents, easy-to-read forms, or visual aids, despite evidence that these tools improve comprehension and health outcomes [36]. Moreover, the absence of standardized communication protocols across healthcare systems perpetuates fragmented care and inconsistent accommodation practices, widening disparities for individuals with IDD [37,38].

#### 4.1.3. Policy Barriers

Policy barriers represent a structural determinant that systematically constrains access to healthcare and social services for individuals with IDD. Findings from this review indicate that inequities are not primarily driven by the absence of policy, but rather by inconsistent enforcement, limited implementation, and inadequate accountability within existing legal and regulatory frameworks. Despite the presence of disability rights legislation and accommodation mandates, individuals with IDD continue to encounter barriers that restrict program participation and limit access to essential services [39,40]. At the systems level, policy barriers manifest through insufficient resource allocation for personal assistance services, fragmented coverage structures, and policies that fail to adequately account for the complexity and continuity of care required by individuals with IDD. These shortcomings often result in exclusion from federally funded programs, reduced access to benefits, and limited opportunities for social inclusion. A critical contributor to these disparities is the lack of awareness, monitoring, and enforcement of existing accessibility requirements, which allows noncompliance to persist across healthcare and social service settings [41]. Policy barriers are closely intertwined with financing and insurance structures. Navigating complex public insurance systems, particularly Medicaid, poses substantial challenges for individuals with IDD and their caregivers. Inadequate reimbursement rates discourage provider participation and limit service availability, while the higher prevalence of chronic and co-occurring conditions among individuals with IDD increases both care complexity and cost [42]. Consequently, policy design and payment mechanisms often determine whether individuals with IDD can access timely, comprehensive, and coordinated care. Consistent with previous research, Pham et al. [43] noted the need for policy reforms, including expanding Medicaid dental and vision benefits, greater integration of physical and mental healthcare services, and sustained investment in data systems to support person-centered care and monitor disparities. However, this review emphasizes that policy reform alone is insufficient. Without effective implementation strategies, even well-designed policies may fail to achieve equitable outcomes. Limited provider awareness of legal obligations, weak institutional accountability, and variability in policy application across settings continue to contribute to persistent inequities in healthcare access and health outcomes for individuals with IDD [44,45].

#### 4.1.4. Programmatic Barriers

This narrative review identified programmatic barriers as a central contributor to gaps in healthcare quality and access for individuals with IDD. These barriers arise from the structure, policies, and operational features of healthcare programs that inadvertently exclude or inadequately support this population. Across the literature, rigid scheduling practices, insufficient appointment time, and inflexible clinical procedures consistently constrained meaningful participation in care for individuals with IDD. Programs that do not allow extended visit times, alternative scheduling options, or preparatory supports limit opportunities for symptom disclosure, shared decision-making, and engagement in preventive care [24,39,46]. Such constraints contribute to delayed or missed diagnoses, reduced uptake of screening services, and lower-quality clinical encounters, ultimately worsening health outcomes for this population [46–48]. Programmatic barriers are further reinforced by inadequate provider training, fragmented service delivery, inflexible care models, and inaccessible communication practices. Numerous studies report that healthcare providers often lack the training and confidence necessary to effectively care for individuals with IDD, resulting in the avoidance of complex cases, shortened visits, and compromised care quality [13,24]. Importantly, these challenges reflect systemic gaps in medical education, continuing professional development, and organizational support rather than individual provider shortcomings. Healthcare systems frequently rely on standardized service delivery models that fail to accommodate the cognitive, communication, and support needs of individuals with IDD. Short appointment times, rigid workflows, and limited accommodation for caregiver involvement hinder meaningful patient–provider interactions and reduce the effectiveness of clinical encounters [49,50]. This lack of flexibility serves as a programmatic barrier, systematically excluding individuals who require adapted care processes. Programmatic inadequacies also contribute to inefficient patterns of healthcare utilization. Individuals with IDD experience higher rates of avoidable hospitalizations and emergency department use, often for conditions that could be managed in primary care settings with appropriate preventive and supportive services [51]. These patterns reflect failures in care coordination and preventive care delivery rather than increased clinical need alone. Consistent with these findings, prior studies indicate that programmatic inequities are reinforced by the absence of disability-competent practices within healthcare systems. When programs do not mandate disability-specific training, standardized communication supports, or accessible patient education materials, individuals with IDD are more likely to encounter fragmented and inconsistent care [36,37]. Additionally, limited integration of interdisciplinary and supportive services, such as behavioral specialists, social workers, and care coordinators, reduces healthcare systems’ capacity to address the complex medical and social needs of individuals with IDD [52]. Without integrated, team-based approaches, care remains siloed, inefficient, and poorly aligned with long-term health management, perpetuating inequities across the continuum of care.

#### 4.1.5. Social Barriers

Social barriers, closely linked to the broader social determinants of health, play a critical role in shaping inequitable outcomes for individuals with IDD. These barriers arise from the social and economic contexts in which individuals with IDD live, including limited educational opportunities, employment challenges, income inequality, and increased exposure to violence. Across the literature, individuals with IDD experience disproportionate exposure to socioeconomic marginalization, characterized by lower educational attainment, limited employment opportunities, and elevated poverty rates [24,53]. These inequities are further compounded among racial and ethnic minority individuals with IDD. Black and Hispanic individuals with IDD face intersecting forms of racial and disability-based discrimination, resulting in lower employment rates, reduced wages, decreased college enrollment, and fewer employment-related benefits compared with their White peers [54]. Importantly, these disparities reflect structural inequities rather than individual limitations. Individuals with IDD are frequently marginalized within labor markets, excluded from competitive employment, or confined to low-wage positions with limited opportunities for advancement [55]. Employment inequities are closely linked to educational disparities, as segregated educational settings and inadequate accommodation restrict skill development, vocational preparedness, and long-term social inclusion [53,56]. Economic disadvantage further amplifies health risks by constraining access to essential resources for well-being. Limited income and reliance on public benefits are associated with reduced access to healthcare services, nutritious food, stable housing, and reliable transportation, factors consistently identified as key determinants of health outcomes for individuals with IDD [57]. Consistent with the findings of this review, prior studies also report that social barriers increase exposure to violence, neglect, and victimization. Individuals with IDD experience significantly higher risks of abuse, driven by social isolation, dependence on caregivers, communication barriers, and limited access to protective and advocacy resources [58,59]. These experiences have profound and lasting implications for both physical and mental health, further entrenching disparities across life.

#### 4.1.6. Physical and Transportation Barriers

Physical barriers within both natural and built environments substantially restrict mobility, independence, and access to essential services for individuals with IDD. Architectural obstacles, such as steps, curbs, narrow doorways, and uneven sidewalks, impede safe navigation and access to public and private spaces for this population [60,61]. Significant healthcare barriers persist for individuals with developmental disabilities, including physical obstacles such as inaccessible clinical facilities and inadequate medical equipment, as well as systemic challenges such as transportation gaps and insufficient insurance coverage. These barriers contribute to incomplete care, missed preventive screenings, and poorer health outcomes, effectively rendering access to basic healthcare services a human rights concern, particularly in resource-limited settings [62]. Within healthcare settings, the lack of accessible medical equipment, such as adjustable examination tables, wheelchair-accessible scales, and adaptable imaging technologies, further restricts participation in preventive and routine care. These structural limitations do more than inconvenience individuals with IDD; they directly contribute to health disparities by limiting service access, reinforcing social exclusion, and increasing reliance on caregivers for physical assistance [49,57]. Consistent with these findings, prior studies have shown that physical mobility challenges significantly hinder engagement in physical activity and overall participation in community life among both children and adults with IDD [63,64].

Transportation barriers significantly exacerbate healthcare inequities for individuals with IDD. Limited access to public transportation, reliance on caregivers or others for travel, long distances to medical facilities, and communication challenges during transit contribute to delayed or missed appointments and inadequate care [65,66]. These barriers disproportionately affect individuals living in rural or underserved areas, further amplifying existing health disparities [36,66]. Unmet transportation needs have been consistently linked to reduced community participation, limited access to essential services, and lower quality of life for individuals with IDD [67,68]. Additionally, individuals with IDD face substantial barriers to using public transportation, including long wait times, high costs, unreliable services, physical obstacles (e.g., stairs and long distances), safety concerns, complex travel planning, and negative attitudes from drivers [69]. These challenges significantly limit the ability to attend medical appointments, access preventive and specialty care, and obtain timely treatment, particularly for individuals who rely on public transit as their primary means of transportation. As a result, transportation barriers contribute to delayed care, missed appointments, and reduced continuity of care, further exacerbating health disparities among individuals with IDD. Consistent with these findings, Wolfe et al. [70] reported that transportation barriers significantly hinder U.S. healthcare access, with millions delaying or skipping care annually, especially impacting low-income adults, people of color (like American Indian/Alaska Natives), individuals with disabilities, and those with chronic conditions, leading to missed appointments and worse health outcomes. Conversely, evidence suggests that reliable and accessible transportation is a critical determinant of health. When individuals with IDD have improved access to transportation, they are more likely to attend medical appointments, receive preventive care, and use timely health services, resulting in better overall health outcomes [71,72]. Overcoming these barriers requires coordinated, multisectoral strategies that integrate accessible transit, transportation assistance programs, and communication supports.

### 4.2. Contributors to Health Disparities Among Individuals with IDD

#### 4.2.1. Limited Healthcare Access

This narrative review identified limited healthcare access as a major contributor to health disparities among IDD, driven by multiple systemic and structural barriers. These barriers include a shortage of healthcare providers trained in IDD-specific care, transportation limitations, inadequate insurance coverage, and persistent communication challenges [36,46,57]. Evidence across the literature indicates that these access barriers have a direct and substantial impact on healthcare experiences and health outcomes for individuals with IDD. Healthcare access is frequently hindered by provider training gaps, inaccessible health information (e.g., complex forms and automated systems), negative provider attitudes, diagnostic overshadowing, and physical and systemic obstacles. Collectively, these factors contribute to poorer health outcomes and reduced quality of care. While emerging efforts, such as enhanced provider education, community-based care models (e.g., START), and reasonable accommodations, aim to improve equity, access to quality and affordable healthcare remains insufficiently realized for this population [73]. Similarly, Bacherini et al. [74] emphasized that health and healthcare are central to the quality of life of individuals with IDD, yet persistent barriers, including inadequate access, limited provider training, and societal discrimination, contribute to higher rates of chronic conditions, preventable morbidity, and premature mortality. Additionally, a cross-sectional study by Stone et al. [75] further reported that adults with cognitive disabilities experienced lower satisfaction with healthcare services than the general population and were less likely to report that providers listened attentively or communicated information clearly. Such deficiencies contribute to delayed preventive care, progression of chronic conditions, and missed opportunities for early intervention, ultimately worsening health outcomes. Consistent with the findings of this review, individuals with IDD experience higher rates of unmet healthcare needs and lower utilization of essential services compared with the general population [57,76]. These inequities in access to care are further exacerbated by broader social determinants of health, including poverty, educational disparities, and social isolation, which collectively constrain access to healthcare resources [8,77]. For example, Ploeg Booth [78] reported that individuals with Down syndrome frequently receive inadequate preventive care and are less likely to access subspecialty services, such as cardiology and gynecology, despite elevated health risks. Similarly, Mudrick et al. [3] documented widespread inaccessibility of medical facilities and equipment, creating additional barriers to routine and preventive care. Disparities in healthcare access are particularly more prevalent among racial and ethnic minority individuals with IDD. Scott and Havercamp [79] found that minority populations, especially Hispanic Americans with IDD, face substantial disadvantages in healthcare utilization and remain among the most underserved groups.

#### 4.2.2. Comorbidity

Comorbidity is a major contributor to health disparities among individuals with IDD and is highly prevalent in this population, with disproportionately higher rates of co-occurring chronic physical and mental health conditions [7,80]. Evidence from a cross-sectional study indicates that adults with intellectual disabilities (ID) experience distinct patterns of chronic disease, often developing conditions such as diabetes, cardiovascular disease, asthma, hypertension, and thyroid disorders at earlier ages and with higher levels of multimorbidity, including mental health conditions, epilepsy, and autism, compared to adults without ID [81]. These co-occurring conditions substantially increase the complexity of healthcare management and contribute to poorer health outcomes relative to the general population [57]. Compared to individuals without disabilities, this population is more likely to have multiple chronic comorbidities (Pastor-Barriuso et al. [82], less likely to engage in regular physical activity [83], and more likely to be overweight or obese [84]. These disparities indicate the intersection of disability, limited access to health-promoting opportunities, and systemic barriers that disproportionately increase the health risks in this population. Across the life course, children with developmental disabilities experience higher rates of diabetes, injury-related hospitalizations, and dental surgery compared to children without disabilities [85,86]. Adults with cognitive limitations similarly face increased burdens of diabetes, asthma, hypertension, arthritis, and stroke [87], as well as elevated risks of nephrolithiasis and other chronic conditions [88,89]. Consistent with these findings, prior studies reported that the high prevalence of comorbidity among individuals with IDD poses substantial challenges for healthcare systems. Multiple chronic conditions complicate diagnosis, treatment planning, and care coordination, often requiring more intensive and specialized management [57]. Importantly, these clinical complexities do not occur in isolation but intersect with other barriers, including limited access to healthcare, communication challenges, and adverse social determinants of health, further exacerbating disparities. Given these multifaceted health needs, integrated and multidisciplinary approaches are essential.

#### 4.2.3. Low Health Literacy

Low health literacy is also a critical factor influencing the health outcomes of individuals with IDD. This challenge undermines the ability of individuals with IDD to understand preventive measures, engage in self-care, and navigate healthcare systems, frequently resulting in delayed care and poorer health outcomes. According to Nguyen & Gilbert [90], individuals with IDD often face significant health literacy challenges, experiencing more effort, frustration, and difficulty understanding health information compared to those without disabilities. Geukes et al. [91] revealed in a mixed-method study that individuals with IDD face significant healthcare challenges due to cognitive/communicative barriers, leading to lower functional health literacy, less involvement in decisions, and worse health outcomes. The author further emphasized the need for a tailored, targeted intervention, particularly for this population. Cognitive and communication impairments can limit how individuals with IDD acquire, process, and apply health-related information [13,92]. As a result, many individuals struggle to understand medical instructions, recognize symptoms that require medical attention, or follow their treatment plans. Low health literacy exacerbates these difficulties. Havercamp and Scott [36] reported that individuals with IDD often encounter significant difficulties in interpreting medical advice, navigating complex healthcare systems, and making informed decisions about their care, which in turn limits engagement in preventive services and exacerbates existing health disparities. These patient-level challenges are further intensified by provider- and system-level barriers, including insufficient physician training, limited experience caring for individuals with IDD, and inadequate appointment time, all of which hinder the delivery of effective and equitable care [93,94]. Such constraints can lead to communication breakdowns, missed clinical cues, and fragmented care. Consistent with these findings, prior studies have documented disparities in preventive, primary, and specialty care for individuals with IDD, many of which stem from clinicians’ lack of disability-specific knowledge and discomfort in treating this population [11,46,95]. Low health literacy among individuals with IDD is exacerbated by the absence of accessible educational materials, plain-language communication, visual supports, and teach-back approaches tailored to cognitive needs. These gaps limit understanding, reduce engagement in care, and impair individuals’ ability to manage their health effectively. Concurrently, inadequate provider training in disability competence, unstructured communication practices, insufficient appointment time, and fragmented care coordination further compound these challenges, leading to poor clinical interactions and suboptimal health outcomes. Together, these patient- and system-level deficits reinforce health disparities and hinder progress toward equitable healthcare for individuals with IDD.

#### 4.2.4. Caregivers Burden

This narrative review identified caregiver burden as a significant contributor to health disparities among individuals with IDD, given that caregiver well-being directly influences the quality, consistency, and effectiveness of care. Caring for individuals with intellectual disabilities is often demanding, as caregivers experience physical, emotional, financial, and social challenges, collectively referred to as caregiver burden [96]. This burden is multifaceted and shaped by interrelated factors, including the severity of the individual’s disability, co-occurring mental or behavioral conditions, availability of social support, and caregivers’ coping capacity [97,98]. Caregivers, whether family members or paid support staff, frequently manage complex medical, behavioral, and social needs with limited resources, placing them at high risk for stress, emotional exhaustion, and burnout. This strain can limit caregivers’ capacity to support individuals with IDD in accessing and navigating healthcare services [96,99,100]. Family caregivers, in particular, experience disproportionately higher burden, with elevated rates of depression and anxiety driven by sustained emotional, physical, and financial demands and social isolation, often exceeding those observed in the general population and, in some cases, the care recipients themselves [101]. Dawson et al. [102] and Irazábal et al. [103] reported that greater functional impairment and the presence of psychiatric or behavioral problems are strongly associated with higher perceived caregiver burden. Care coordination further compounds these challenges. Doherty et al. [11] noted that even clinicians often feel overwhelmed when caring for individuals with IDD, suggesting that caregivers with fewer resources and less formal training experience even greater strain. Caregivers of children with neurodevelopmental disorders face significant barriers navigating fragmented health, education, and social service systems, where care is often diagnosis-specific rather than holistic. This fragmentation contributes to uncoordinated care, caregiver burnout, and compromised family well-being [104,105]. These findings align with existing evidence; a cross-sectional study by Barik et al. [106] found that family caregivers of children with autism spectrum disorder experienced substantial burden, particularly subjective internalized burden, which was strongly associated with lower educational attainment, lower socioeconomic status, and nuclear family structures. Collectively, these cumulative pressures disrupt continuity of care and further exacerbate health disparities among individuals with IDD.

#### 4.2.5. Social Determinants of Health

Social determinants of health (SDOH) significantly impact health outcomes for individuals with IDD by influencing access to resources, quality of life, nutrition, and overall well-being [107]. Individuals with intellectual disabilities experience greater exposure to adverse social determinants of health, including poverty, unemployment, poor housing, social isolation, and both interpersonal and systemic discrimination [108,109]. According to McKinney et al. [110], lower socioeconomic status (SES) is associated with lower cognitive outcomes, including IQ, largely due to reduced access to essential resources, nutritious food, stimulating environments, and quality educational opportunities. Children with developmental disabilities experience substantially higher rates of unmet health-related social needs (HRSN), including housing instability, food insecurity, healthcare affordability challenges, and legal needs, compared to children without disabilities [9]. Approximately 30% of children with DD report at least one unmet HRSN, a rate nearly 2.5 times higher than their peers [9]. Kersey et al. [111] reported that people with IDD who have low incomes, food insecurity, housing instability, and limited ability to work are more likely to have severe psychological distress. These social and economic challenges significantly increase vulnerability to poor mental health outcomes. A study from the National Health Interview Survey (NHIS) data shows that adults with developmental disabilities experience poorer health outcomes and higher rates of chronic conditions than adults without disabilities. They also face significant barriers to care, largely linked to socioeconomic factors, including lower educational levels, higher poverty rates, and reliance on public insurance [112]. Similar to these findings, other existing research indicates that individuals with IDD face significant disparities in education, income, social support, and access to and use of healthcare services [24,113,114]. Furthermore, social isolation further exacerbates health disparities. Many individuals with IDD have smaller social networks and fewer opportunities for community inclusion, resulting in diminished emotional support, reduced participation in health-promoting activities, and increased vulnerability to neglect or exploitation [35]. Understanding the influence of these social determinants is essential in addressing the broader context of health disparities among individuals with IDD.

## 5. Conclusions

This study emphasizes the complex and interrelated barriers that individuals with intellectual and developmental disabilities face in accessing equitable healthcare. Attitudinal, communication, policy, programmatic, social, physical, and transportation barriers collectively contribute to persistent health disparities. Additional contributors, including limited healthcare access, low health literacy, comorbid conditions, adverse social determinants, and caregiver burden, further exacerbate these inequities. Addressing these challenges requires comprehensive, multi-level strategies, including inclusive policies, tailored communication approaches, provider training, accessible infrastructure, and support for social determinants of health. Implementing such interventions is crucial to promoting health equity, enhancing healthcare access, and improving outcomes for individuals with intellectual and developmental disabilities. Future research should continue to explore effective interventions, evaluate their impact, and provide actionable guidance for policymakers, healthcare providers, and communities to ensure the well-being of this underserved population.

## 6. Implications of the Study

The findings of this study show the urgent need for early, coordinated, and equity-focused interventions to address health disparities among children, adolescents, as well as adults with IDD across the life course. By demonstrating that health inequities stem from the intersection of social, structural, and healthcare-related barriers, rather than individual-level factors alone, this review emphasizes the importance of addressing social determinants of health during critical developmental periods.

From a practice perspective, healthcare systems should integrate disability-informed, family-centered care models that address unmet health-related social needs, caregiver burden, and accessibility challenges. Strengthened provider training, improved care coordination, and developmentally appropriate services are essential for enhancing access to and quality of care for children and adolescents with IDD.

From a policy perspective, these findings emphasize the need for policies that expand access to affordable healthcare, transportation, housing stability, and educational support for families of children with IDD. Enhanced cross-sector collaboration among healthcare, education, and social service systems may reduce fragmentation and promote more equitable outcomes.

## 7. Limitations of the Study

This narrative review has limitations that should be acknowledged. Being a narrative review, this study may be limited by potential selection bias, which could influence the scope of the findings. Despite using comprehensive search strategies and relevant terms, relevant studies may have been unintentionally omitted despite efforts to capture a broad range of literature. Publication bias is a potential concern, as studies with significant or positive findings are more likely to be published. Additionally, restricting the review to English-language studies may have introduced language bias and excluded relevant literature published in other languages. Finally, the narrative review predominantly reflects findings from high-income countries, which may limit generalizability to low- and middle-income settings where healthcare systems and social contexts differ substantially.

## 8. Future Research

Future research should prioritize longitudinal and intersectional approaches to better understand how these barriers compound over time, particularly among racially and socioeconomically marginalized individuals with IDD. Strengthening data systems, investing in disability-competent care models, and centering the voices of individuals with IDD and their caregivers are essential steps toward advancing health equity and improving population-level outcomes for this underserved group.

## Data Availability

No new data were generated or analyzed in this narrative review. All data supporting the findings are included in the published literature cited in this article.

## Abbreviations

The following abbreviations are used in this manuscript:

IDD: Intellectual and developmental disabilities
DD: Developmental disabilities
SDOH: Social Determinants of Health
